# Lumen‐apposing metal stents for anastomosis creation throughout the gastrointestinal tract: A large single‐center experience

**DOI:** 10.1002/deo2.419

**Published:** 2024-10-12

**Authors:** Emine Gökce, Lindsey Devisscher, Niki Rashidian, Enrico Palmeri, Pieter Hindryckx

**Affiliations:** ^1^ Department of Gastroenterology and Hepatology Ghent University Hospital Ghent Belgium; ^2^ Department of Basic and Applied Medical Sciences Gut‐Liver Immunopharmacology Unit, Ghent University Ghent Belgium; ^3^ Department of Liver Research Center Ghent Ghent University Ghent Belgium; ^4^ Department of HPB Surgery and Liver Transplantation Ghent University Hospital Ghent Belgium

**Keywords:** EDGE, EDGI, gastric outlet obstruction, lumen‐apposing metal stent, Roux‐en‐Y gastric bypass

## Abstract

**Objectives:**

The introduction of lumen‐apposing metal stents (LAMSs) has revolutionized the field of therapeutic endoscopic ultrasound. This study aims to evaluate the efficacy and safety of LAMS in creating an endoscopic ultrasound‐guided anastomosis between two segments of the gastrointestinal (GI) tract.

**Methods:**

Data from all consecutive LAMS procedures for anastomosis creation between two segments of the GI, conducted between October 2019 and February 2024, were retrospectively analyzed for technical success (defined as correct deployment of the LAMS in the target), clinical success (defined as achievement of the intended clinical goal), and adverse events.

**Results:**

A total of 145 LAMS procedures were performed in 136 patients. Indications for LAMS procedures included the need for endoscopic access to or reversal of surgically excluded segments of the GI tract (*n* = 73, 50.3%), and the alleviation of any GI outflow obstruction (*n* = 72, 49.7%). The overall technical and clinical success rates were very high (97.2% and 95.2%, respectively). Adverse events were observed in 20/145 (13.8%) cases, including 11 (7.6%) minor events (AGREE <3) and nine (6.2%) major events (AGREE ≥3). Major events included stent migration (*n* = 1), persisting fistula (*n* = 3), and bleeding (*n* = 4). All adverse events were successfully managed, and there were no procedure‐related deaths. Loss of LAMS patency occurred in 4/145 (2.8%) cases and could be endoscopically managed in all cases.

**Conclusions:**

The creation of anastomoses with LAMS between two segments of the GI tract appears to be effective and safe, with a low reintervention rate due to loss of LAMS patency.

## INTRODUCTION

The introduction of lumen‐apposing metal stents (LAMSs) was an important contribution to the field of therapeutic endoscopic ultrasound (EUS). LAMSs are fully covered, dumbbell‐shaped stents with a short length and a large internal diameter. While they were developed for drainage of pancreatic fluid collections in 2013, they are now also approved for gallbladder drainage in inoperable patients and biliary drainage in malignant distal biliary obstruction and/or failed endoscopic retrograde cholangiopancreatography (ERCP).^1^, ^2^, ^3^

**FIGURE 1:**
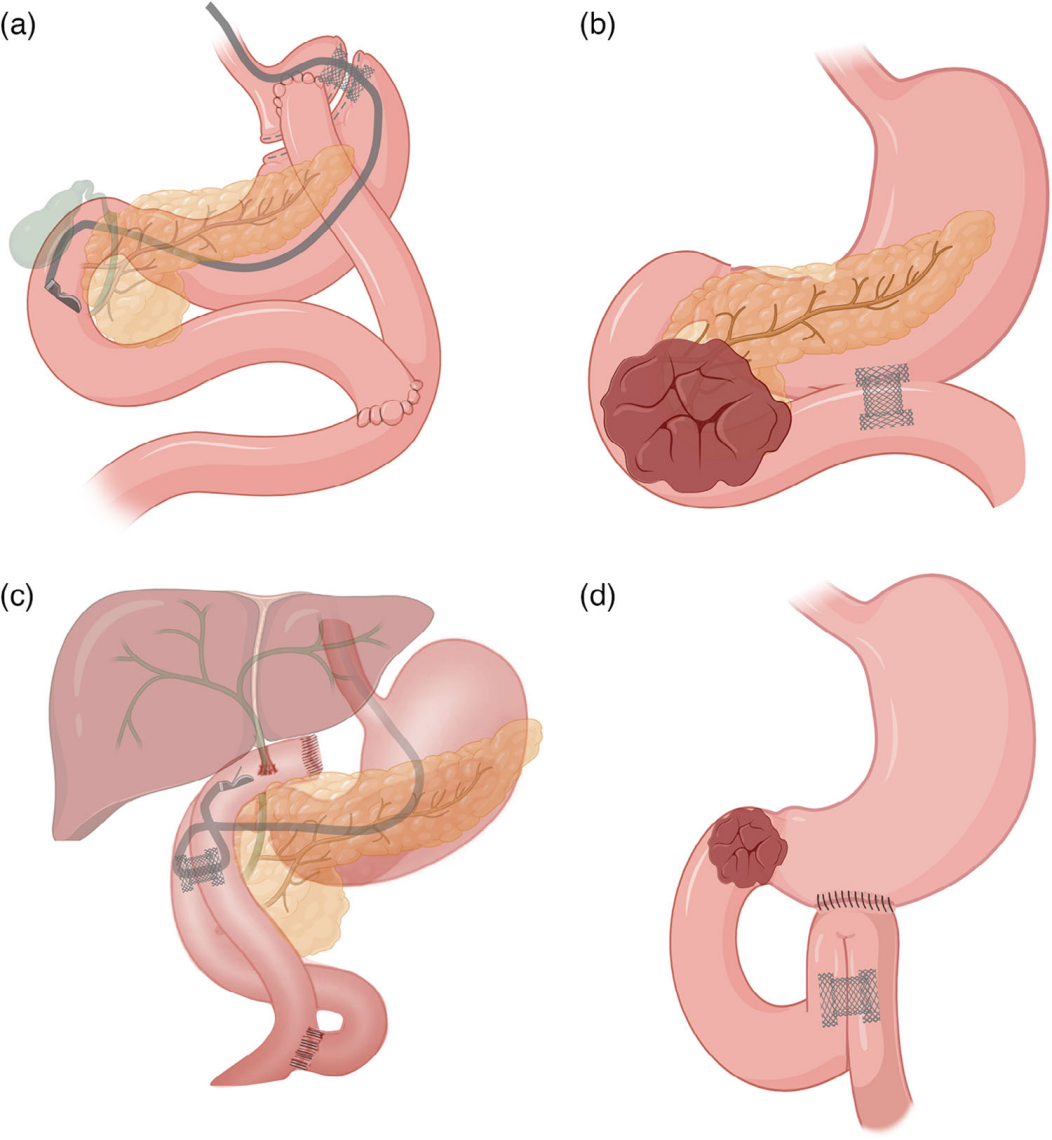
Main types of lumen‐apposing metal stent anastomosis throughout the gastrointestinal tract. (a) Endoscopic ultrasound‐guided gastro‐gastrostomy after Roux‐en‐Y gastric bypass. (b) Endoscopic ultrasound‐guided gastro‐enterostomy in unaltered anatomy. (c) Endoscopic ultrasound‐guided duodeno‐enterostomy after surgical hepaticojejunostomy. (d) Endoscopic ultrasound‐guided entero‐enterostomy for afferent loop syndrome after gastrojejunostomy surgery.

The use of LAMSs for off‐label indications has increased significantly and now accounts for about 50% of LAMS use in referral centres^4^. The unique bi‐flanged design of LAMSs offers the ability to hold two luminal structures in apposition and therefore allows for anastomosis creation between two adjacent segments of the gastrointestinal (GI) tract. The most commonly created anastomoses are displayed in Figure 1).^1^, ^2^, ^3^, ^4^

Indications for anastomosis creation include: (1) the need for endoscopic access to excluded parts of the GI tract in patients with surgically altered anatomy, (2) alleviation of malignant or benign gastric or enteral outlet obstruction, and (3) a medical need for reversal of a surgical bypass.

The aim of this study was to evaluate the performance of LAMS for anastomosis creation throughout the GI tract.

## METHODS

This study was reviewed and approved by the Ethics Committee of Ghent University Hospital, Belgium (reference number ONZ‐2024‐0218).

### Data collection

All consecutive patients who underwent EUS‐guided gastro‐gastrostomy (EUS‐GG), gastro‐enterostomy (EUS‐GE), or entero‐enterostomy (EUS‐EE) between October 2019 and February 2024 at Ghent University Hospital were included. The data were retrieved from a prospectively maintained LAMS database (containing all consecutive patients) and missing data were retrieved from a review of the electronic medical patient records. We divided the patients into two main groups: (1) patients with surgically altered anatomy requiring endoscopic access to (1a) or reversal of (1b) an excluded GI segment and (2) patients with any GI outflow obstruction including malignant gastric outlet obstruction (mGOO; 2a), benign gastric outlet obstruction (bGOO; 2b), candy cane syndrome (2c), or afferent loop syndrome (2d).

The main outcome data of interest were technical success, clinical success, and adverse events. Technical success was defined as a successful placement of LAMS in the desired position. Clinical success was defined as either successful endoscopic access to the intended excluded gastrointestinal segment in patients with surgically altered anatomy (for subcategory 1a), successful re‐initiation of oral food intake (for subcategories 2a and 2b), or achievement of the intended therapeutic goal for subcategories 1b, 2c and 2d. Adverse events were classified according to the AGREE criteria and grouped into minor (AGREE I and II) and major adverse events (AGREE IIIa, IIIb, and IV).^5^

### Procedural techniques

Procedures were performed by highly trained interventional endoscopists with expertise in ERCP and interventional EUS. Only Hot Axios stents (Boston Scientific) were used.

#### EUS‐guided gastro‐gastrostomy

The excluded stomach was identified with EUS from within the stomach pouch. A 19G needle was used to puncture the lumen of the excluded stomach. After fluoroscopic confirmation of the correct position of the needle tip, stained fluid was instilled in the excluded stomach until sufficient space was created for the placement of a 15/10 or 20/10 LAMS. For non‐urgent indications, endoscopic intubation of the LAMS lumen was performed after an interval of at least 7 days, to allow for fistula tract maturation and thereby reduce the risk of perforation upon dislocation of the LAMS. For urgent indications (e.g., severe cholangiosepsis), endoscopic intubation of the excluded stomach was performed in the same procedure after fixation of the LAMS and balloon dilatation of the LAMS lumen up to 18 mm (Video S1).

#### EUS‐guided gastro‐enterostomy

Using a therapeutic gastroscope with a working channel of 3.7 mm, a guidewire was placed in the proximal jejunum, right after the angle of Treitz. A 7 Fr nasobiliary drain was positioned over the wire, right distal from the angle of Treitz, and the jejunal segment was filled with indigo carmine‐stained water. The target loop was identified within the stomach with EUS. When sufficient distension of the jejunal segment was reached, a 20/10 LAMS was placed. The correct position was verified with contrast enterography through the LAMS lumen (Video S2).

#### EUS‐guided entero‐enterostomy

Through the lumen of the duodenum or the efferent enteral limb, the afferent enteral limb was identified with EUS and punctured with a 19 G needle. The correct position was verified with contrast enterography. The jejunal segment was filled with stained water. When sufficient distension of the jejunal segment was reached, a 15/10 or 20/10 LAMS was placed. The correct position was verified with contrast enterography through the LAMS lumen (Video S3).

## RESULTS

### Patient characteristics

A total of 145 LAMS were placed in 136 patients (mean age, 60 years; 62.5% women) to create an EUS‐GG, EUS‐GE, or EUS‐EE anastomosis. Baseline patient characteristics per indication are presented in Table 1.

**TABLE 1:** Patient characteristics and procedural details by indication (surgically altered anatomy access/reversal versus gastro‐intestinal outflow obstruction) and overall.

Indication (*n* = X)	Access/reversal surgically altered GI tract (*n* = 73)	GI outflow obstruction (*n* = 72)	Total (*n* = 145)
**Patient characteristics**			
Indications	Need for ERCP, *n* = 55 (75.3%)	Alleviation benign GOO, *n* = 19 (26.4%)	
	Need for EUS+FNA, *n* = 5 (6.8%)	Alleviation malignant GOO, *n* = 50 (69.4%)	
	Need for gastroduodenoscopy, *n* = 8 (11.0%)	Afferent loop syndrome, *n* = 2 (2.8%)	
	Undo of gastric bypass, *n* = 5 (6.8 %)	Candy cane syndrome, *n* = 1 (1.4%)	
Mean age (years)	54	67	60
Female, *n* (%)	54 (74.0%)	37 (51.4%)	91 (62.8%)
ASA			
ASA I, %	5.4	1.9	3.6
ASA II, %	58.9	33.3	56.4
ASA III, %	35.7	64.8	50.0
Underlying malignancy, *n* (%)	4 (5.5%)	51 (70.8%)	55 (37.9%)
Non‐procedure related death, *n* (%)	5 (6.8%)	38 (52.8%)	43 (29.7%)
**Procedural details**			
Type of LAMS anastomosis			
EUS‐GG, *n* (%)	70 (95.9%)	0	70 (48.3%)
EUS‐GE, *n* (%)	0	70 (97.2%)	70 (48.3%)
EUS‐EE, *n* (%)	3 (4.1%)	2 (2.8%)	5 (3.4%)
LAMS diameter/length, mm			
15/10	6 (8.2%)	0	6 (4.1%)
20/10	67 (91.8%)	72 (100%)	139 (95.9%)
LAMS dilatation, *n* (%)	27 (37.0%)	34 (47.2%)	61 (42.1%)
LAMS fixation, *n* (%)	36 (49.3%)	33 (45.8%)	69 (47.6%)
Dual session procedure	51 (69.9%)	n/a	n/a
Median duration of LAMS placement, min	29 (min 5; max 105; mean 35)	36 (min 14; max 122; mean 40)	31 (min 5; max 122; mean 35)

Abbreviations: ERCP, endoscopic retrograde cholangiopancreatography; EUS, endoscopic ultrasound; EUS‐EE, endoscopic ultrasound‐guided entero‐enterostomy; EUS‐GE, endoscopic ultrasound‐guided gastro‐enterostomy; EUS‐GG, endoscopic ultrasound‐guided gastro‐gastrostomy; FNA, fine needle aspiration, GI, gastrointestinal; GOO, gastric outlet obstruction; LAMS, lumen‐apposing metal stents

The most common indication for anastomosis creation was the need for (temporary) access to an excluded part of the GI tract (68/145; 46.9%). Most patients (65/68; 95.6%) had a previous Roux‐en‐Y Gastric Bypass (RYGB) and needed access to the excluded stomach or duodenum. The main reason was the need for EUS‐directed transgastric ERCP (EDGE procedure; 55/65 cases; 84.6%).

The second most frequent indication was the alleviation of gastric outlet obstruction (GOO; 69/145; 47.6%), mostly in the setting of malignancy (50/69; 72.5%). Cases of benign gastric outlet obstruction (19/69; 27.5%) were mainly related to chronic pancreatitis with groove components (*N* = 9/19; 47.4%).

Further, five EUS‐GG were performed in patients who needed a reversal of RYGB, including one cachectic patient due to metastatic cholangiocarcinoma, three patients with hyperinsulinemic hypoglycemia after RYGB (included in an ongoing in‐house study), and one patient with therapy‐resistant small intestinal bacterial overgrowth (SIBO). Lastly, two patients received an LAMS for treatment of an afferent loop syndrome post‐Whipple surgery, and one patient to manage a candy cane syndrome.

### Procedural outcomes

#### Technical and clinical success

The overall technical and clinical success rate was very high (141/145 [97.2%] and 138/145 [95.2%], respectively). Success rates per indication and other procedural outcomes are shown in Table 2.

**TABLE 2:** Procedural outcomes by indication (surgically altered anatomy access/reversal vs. gastro‐intestinal outflow obstruction).

Indication (*n* = X)	Access/reversal surgically altered GI tract (*n* = 73)	GI outflow obstruction (*n* = 72)	Total (*n* = 145)
Technical success, *n* (%)	71 (97.3%)	70 (97.2%)	141 (97.2%)
Clinical success, *n* (%)	70 (95.9%)	68 (94.4%)	138 (95.2%)
Adverse events, *n* (%)	11 (15.1%)	9 (12.5%)	20 (13.8%)
Minor (AGREE I, II)	7 (9.6%)	4 (5.6%)	11 (7.6%)
Major (AGREE IIIa, IIIb, IV)	4 (5.5%)	5 (6.9%)	9 (6.2%)
Procedure‐related deaths (*n*)	0	0	0
Endoscopic LAMS removal, *n* (%)	53 (72.6%)	5 (6.9%)	58 (40.0%)
Median LAMS dwell time (days)	38 (min 7; max 431; mean 61)	86 (min 7; max 602; mean 140)	67 (min 7; max 602; mean 67)
Median follow‐up, days	120 (min 10; max 683; mean 165)	134 (min 7; max 602; mean 197)	106 (min 7; max 683; mean 165)
Loss of patency (all procedures)	1 (1.4%)	3 (4.2%)	4 (2.8%)

Abbreviations: AGREE, adverse events in GI endoscopy; GI, gastrointestinal; LAMS, lumen‐apposing metal stents.

Four clinical failures were related to technical failure. One patient in the mGOO subgroup could not reinitiate oral intake despite a correct LAMS position. One patient with afferent loop syndrome in whom cholestasis did not improve after LAMS placement needed surgical intervention. The last clinical failure was seen in a patient with cachexia in metastatic cholangiocarcinoma, in whom partial breakdown of her gastric bypass with a LAMS did not improve her nutritional status.

### Adverse events

Adverse events were seen in 20/145 cases (13.8%; from which 11 [7.6%] were minor and nine were [6.2%] major) after a median follow‐up duration of 106 days (range 7–683). The adverse event rate, management, and clinical outcome per indication are provided in Table 3. With the exception of the persistent fistula, all complications manifested within the 30‐day post‐procedural period. There was no procedure‐related mortality.

**TABLE 3:** Adverse events by indication (surgically altered anatomy access/reversal versus gastrointestinal outflow obstruction).

Indication	Adverse event	Complication	Needed action	Outcome
Access/reversal surgically altered GI tract (*n* = 11)	Minor (*n* = 7)	Abdominal pain/nausea/vomiting (*n* = 5)	Medication	Complete recovery
		Bleeding (*n* = 2)	One CT abdomen; one observation	Complete recovery
	Major (*n* = 4)	Stent migration (*n* = 1)	Surgery	Complete recovery
		Respiratory desaturation (*n* = 1)	Observation PACU	Complete recovery
		Persisting fistula (*n* = 2)	One surgery; one endoscopy	Complete recovery
GI outflow obstruction (*n* = 9)	Minor (*n* = 4)	Abdominal pain/nausea (*n* = 4)	Medication	Complete recovery
	Major (*n* = 5)	Bleeding (*n* = 4)	Endoscopy	Complete recovery
		Refractory enterocolonic fistula (*n* = 1)	Endoscopy	Complete recovery

Abbreviation: GI, gastrointestinal; PACU, Post Anesthesia Care Unit.

### Loss of patency

Indwelling times of LAMSs ranged from 7 to 602 days. Loss of LAMS patency necessitating endoscopic reintervention was observed in 4/145 cases (2.8%) after a median interval of 85 days (range 39–592). Patency could be endoscopically restored in all cases.

## DISCUSSION

In this large cohort study, LAMSs performed well for anastomosis creation in the GI tract, with a high overall technical (97.2%) and clinical (95.2%) success rates and an acceptable rate of adverse events (13.8%).

In our study, within the subgroup of patients with RYGB, a LAMS was placed for three indications: EDGE, EUS‐directed transgastric intervention, and a medical need for the reversal of RYGB.

EUS‐directed transgastric intervention and EDGE have become accepted approaches to gain access to the excluded upper GI tract and deal with pancreaticobiliary disorders in patients with previous gastric bypasses. Our outcome data align with previous reports.^6^, ^7^, ^8^

In almost 70% of cases, EDGE is performed with a dual‐step approach, to allow for fistula tract maturation and reduce the risk of perforation. The advent of reliable stent fixation devices may change this practice and reduce the burden for the patients.^9^

When access to the excluded GI segment is no longer necessary, we remove the LAMS and close the fistula tract, to minimize the risk of a persisting fistula and resulting weight gain. Although the available data suggest a high rate of spontaneous fistula closure,^10^ we experience that most patients demand closure themselves to maximally reduce the potential risk of a persisting fistula and the resulting weight gain. Nevertheless, two persisting fistulas were seen in our cohort, of which one was surgically closed in the referring center.

Although the numbers are small, we observed promising results of LAMS placement for partial breakdown of a gastric bypass, especially for the indication of hyperinsulinemic hypoglycemia. In this regard, we anticipate that the ongoing LABOR study (NCT05640947) at our unit will provide further insights into effectiveness, safety, and potential indications.

Furthermore, one patient with therapy‐resistant SIBO after RYGB experienced significant improvements in diarrhea and bloating and a (desired) weight gain of 9 kg after LAMS placement. Further research is needed to validate these findings.

Among patients with gastrointestinal outflow obstruction, mGOO was the most common indication for LAMS placement. Both our study and existing literature report high success and acceptable adverse event rates.^4^, ^11^, ^12^, ^13^, ^14^, ^15^ EUS‐GE for mGOO is mainly performed in a palliative setting. EUS‐GE offers superior functional outcomes and a reduced rate of reintervention compared to traditional duodenal stents.^14^, ^16^, ^17^ In addition, the available literature suggests shorter recovery time, lower adverse event rates, and reduced costs compared to surgical gastroenterostomy.^14^, ^15^ Three randomized controlled trials are currently ongoing to compare EUS‐GE with surgical GE: EATING trial (NCT05605327), GOOSE trial (tNCT06071507), and ENDURO trial (ICTRP: NL9592). These studies will possibly confirm EUS‐GE as the preferred approach to manage malignant gastric outlet obstruction in the palliative setting.

Our subgroup of patients with bGOO includes cases such as benign duodenal stenosis resulting from caustic injury, ulcers, pancreatitis, etc. Reports from the literature suggest that the placement of LAMS can prevent surgery in the majority of cases.^18^ Our results were consistent with the literature. We achieved a technical success rate of 94.7% and a clinical success rate of 89.5% respectively. The European Society of Gastrointestinal Endoscopy guidelines recommend the removal of LAMS when the underlying disease is managed to prevent stent site ulceration. In our cohort, it was possible to remove the LAMS (without closure of the fistula tract) in 4/19 patients (21.1%) after a median interval of 85 days [range 51–134], without relapse of GOO. In only 1/19 patients, a loss of patency was observed over time and endoscopically managed. In all other patients, long‐term indwelling of the LAMS was uneventful. Similar to our own and others’ experience in patients with mGOO, the patency of the LAMS seems to be unaffected as long as the gastric outlet obstruction persists (and hence, the created EUS‐GE is still in use).^19^

Nevertheless, further research is essential to precisely define EUS‐GE's role and safety in bGOO.

LAMS placement for other outflow obstruction conditions, such as afferent loop syndrome and candy cane syndrome, remains experimental with limited evidence. Nonetheless, it may offer a less invasive alternative for patients who are not fit for surgery.^20^, ^21^

In conclusion, our study shows that EUS‐GG, EUS‐GE, and EUS‐EE with LAMSs offer an effective and safe approach to creating anastomoses between gastrointestinal segments. The inclusion of less common indications, the large sample size, and the extended follow‐up duration enhance the reliability of our findings. Limitations of our study are the retrospective study design without control over confounding factors and the fact that all procedures were performed by highly trained endoscopists in a high‐volume tertiary center. This might limit the generalization of our findings.

Data from prospective studies are required to formally approve the indications for LAMS listed in our paper.

## CONFLICT OF INTEREST STATEMENT

Pieter Hindryckx has received speaker and/or consultancy fees from Boston Scientific, Taewoong Medical, Duomed, and Viatris. Lindsey Devisscher, Niki Rashidian, Enrico Palmeri, and Emine Gökce declare no conflict of interest.

## ETHICS STATEMENT

This study was reviewed and approved by the Ethics Committee of Ghent University Hospital, Belgium (reference number ONZ‐2024‐0218).

## Supporting information

TABLE S1 Classification for adverse events in GI endoscopy: the AGREE classification.

VIDEO S1 Endoscopic ultrasound‐guided gastro‐gastrostomy (EUS‐GG).

VIDEO S2 Endoscopic ultrasound‐guided gastro‐enterostomy (EUS‐GE).

VIDEO S3 Endoscopic ultrasound‐guided entero‐enterostomy (EUS‐EE).
