# Supplementary material for: Lumen‐apposing metal stents for anastomosis creation throughout the gastrointestinal tract: A large single‐center experience
Source: DEN Open. 2024 Oct 12;5(1):e419. doi: 10.1002/deo2.419 (PMC11470743; doi:10.1002/deo2.419)
Supplement: Supplementary file 1 — TABLE S1 Classification for adverse events in GI endoscopy: the AGREE classification. [file DEO2-5-e419-s002.docx]

Supplementary table 1

| **Supplementary table 1. Classification for adverse events in GI endoscopy: the AGREE classification** | |
| --- | --- |
| **Grading** | **Definition** |
| **Grade I** | Adverse events with any deviation of the standard postprocedural course, without the need for pharmacologic treatment or endoscopic, radiologic, or surgical interventions. |
|  | Presentation at the emergency ward, without any intervention or |
|  | Hospital admission (<24 hours), without any intervention or |
|  | Allowed therapeutic regimens are drugs as antiemetics, antipyretics, analgesics, and electrolytes or |
|  | Allowed diagnostic tests: radiology and laboratory tests |
| **Grade II** | Adverse events requiring pharmacologic treatment with drugs other than those allowed for grade I adverse events (ie, antibiotics, antithrombotics, etc) or |
|  | Blood or blood product transfusions or |
|  | Hospital admission for more than 24 hours |
| **Grade III** | Adverse events requiring endoscopic, radiologic, or surgical intervention |
| **Grade IIIa** | Endoscopic or radiologic intervention |
| **Grade IIIb** | Surgical intervention |
| **Grade IV** | Adverse events requiring intensive care unit/critical care unit admission |
| **Grade IVa** | Single-organ dysfunction (including dialysis) |
| **Grade IVb** | Multiorgan dysfunction |
| **Grade V** | Death of the patient |

Adapted from Nass, Karlijn J et al. “Novel classification for adverse events in GI endoscopy: the AGREE classification.” Gastrointestinal endoscopy vol. 95,6 (2022).
